# Novel Genotype Definition and the First Epidemiological Investigation of Canine Adenovirus Type 2 in Dogs in Central China

**DOI:** 10.3389/fvets.2020.00534

**Published:** 2020-08-19

**Authors:** Jun Ji, Wanyu Li, Wen Hu, Xin Xu, Yunchao Kan, Lunguang Yao, Yingzuo Bi, Qingmei Xie

**Affiliations:** ^1^Henan Provincial Engineering Laboratory of Insects Bio-Reactor, Henan Provincial Engineering and Technology Center of Health Products for Livestock and Poultry, Henan Provincial Engineering and Technology Center of Animal Disease Diagnosis and Integrated Control, Nanyang Normal University, Nanyang, China; ^2^College of Animal Science, South China Agricultural University, Guangzhou, China

**Keywords:** canine adenovirus type 2, co-infection, mutation, phylogeny, novel genotype

## Abstract

Infections caused by canine adenovirus (CAdV) type 1 have been reported worldwide in the past two decades. However, only few studies have specifically reported the prevalence of CAdV type 2 (CAdV-2). The present study investigated the persistent circulation of CAdV-2 in dogs with diarrhea in the Henan, Hubei, and Jiangsu provinces in central China from 2017 to 2019. We conducted polymerase chain reaction for detecting CAdV-2 and other related pathogens in 224 rectal swabs of pet dogs and the co-infection of canine diseases was also analyzed. In addition, the structural protein genes—Fiber, Hexon, and Penton—of the isolated CAdV-2 strains were sequenced and analyzed. The similarity between *Hexon* and *Penton* among the 19 strains was 97.4%, as revealed by sequence alignment. Multiple sequence alignment results showed that the *Fiber* gene sequences of these CAdV-2 strains shared 97.4–99.8% nucleotide and 94.1–99.3% amino acid identity with reference sequences and shared only 79.0–80.5% nucleotide and 77.3–80.5% amino acid identity with the vaccine strain CLL, indicating that Fiber harbored most of the variant sites. Furthermore, pairwise sequence comparisons of *Hexon* of CH-JS-1901 and CH-HN-1801 with that of India2006 revealed a novel genotype. Furthermore, protein model prediction showed that the amino acid mutation of fiber protein in 19 strains was located in the head region, that may cause structural changes on the surface of the fiber protein. These findings are of significance for monitoring the epidemiology of CAdV-2 infection and developing a novel vaccine which contribute to understanding genetic evolution of CAdV-2 in China.

## Introduction

Canine adenovirus (CAdV), a member of the *Mastadenovirus* genus of the Adenoviridae family, is one of the pathogens causing severe viral diseases in dogs worldwide (1). There are three major capsid proteins encoded by CAdV—Fiber, Hexon, and Penton. Adenoviral Fiber protein plays a crucial role in infection by attaching the virus to a specific cell surface receptor, whereas the Penton base is responsible for virus internalization (2). The capsid is icosahedral: and the faces are composed of 240 Hexons, each comprising three identical [Hexon] proteins, and the trimer constitutes the outer sheath of the capsid. Because it contains a specific antigenic determinant, it is also the main protein targeted by the host neutralizing antibody (3).

To date, epidemic reports of CAdVs have been mainly conducted in Italy, France, Britain, Turkey, and North America (4–7), where there are wild animals such as wolves, foxes, skunks, wild dogs, and brown bears (8–13). On contact with the carrier, the virus is transmitted via air, body fluids, and excreta (14).

CAdVs are classified into two types: CAdV type 1 (CAdV-1), which causes canine infectious hepatitis and fox encephalitis, and CAdV type 2 (CAdV-2), which causes infectious laryngotracheitis and enteritis (15). CAdV-2 differs from CAdV-1 in virulence, antigen structure, infection ability, and erythrocyte aggregation range. Dogs infected with CAdV-2 show persistent high fever, cough, and other clinical manifestations, such as mucinous rhinorrhea, tonsillitis, laryngotracheitis, pneumonia, vomiting, and diarrhea (16). CAdV-2 is one of the main pathogens that cause canine viral enteritis. Recently, more studies have reported diarrhea in dogs, and the number of cases of CAdV-2 infection has gradually increased. However, only few studies have focused on the association between CAdV-2 and diarrhea in dogs in clinical diagnosis (17–20).

In 1962, Dithfield isolated the A26 strain (Toronto A26/61) of adenoviruses that caused respiratory tract lesions (laryngotracheitis) but not hepatitis (21). Subsequently, CAdV-2 has become popular worldwide. In 1985, Hamelin confirmed that CAdV-2 can cause not only upper respiratory tract diseases but can also proliferate in the digestive tract epithelium and lead to diarrhea in dogs, which may also be associated with the variation of CAdV-2 (22). In China, CAdV-2 infection was previously reported only in Yunnan, Jilin, and other regions (23). At present, most vaccines used to prevent CAdV infection in China are imported, and the epidemic strains may not be completely homologous with the vaccine strains isolated in foreign countries. Moreover, only few studies have reported the prevalence of adenovirus in most parts of China. Therefore, it is essential to investigate the prevalence, variation, and regularity of CAdV-2 and determine the possible association between CAdV-2 and structural changes in major capsid proteins.

Here we studied the prevalence of CAdV-2 in some provinces in central China, amplified and sequenced the structural protein genes of CAdV-2 strains, and compared the obtained sequences with reference sequences of CAdV-2 strains reported in the literature worldwide. We believe that our study will contribute reliable data for research on developing a canine adenoviral vaccine, which is beneficial for the targeted prevention and treatment of CAdV infection.

## Materials and Methods

### Clinical Sample Collection and Virus Screening

Rectal swabs from 224 dogs with evidence of diarrhea and 164 health dogs were collected from December 2017 to March 2019 in the Henan, Hubei, and Jiangsu provinces of China. The clinical samples were mixed in 1.0 mL of phosphate-buffered saline solution (pH 7.2). Aliquots of 200 μL from the above mixture of raw samples were collected for detecting DNA/RNA viruses. All samples were stored at −80°C to prevent virus deactivation. The infection status and co-infections were assessed by investigating the presence of canine parvovirus 2 (CPV-2), canine distemper virus (CDV), CAdV-1, CAdV-2, canine coronavirus (CRCoV), Canine Circovirus (CACV), and canine bocavirus (CBOV) using polymerase chain reaction (PCR) or real-time PCR, as documented in previous reports (23–26). The positive infection rate (%) was calculated as follows: Positive infection rate of samples (%) = (number of positive samples/total number of samples) × 100.

### DNA Extraction and Gene Amplification

Supernatants from the diluted CAdV-2-positive samples were filtered through a 0.22-μm membrane and inoculated onto a monolayer of Madin–Darby canine kidney cells at 37°C in 5% CO_2_, and this process repeated until cytopathic effects were apparent (27). To detect the presence of other viruses mentioned above, total DNA/RNA from cell culture isolates was extracted using the Viral DNA/RNA kit (TaKaRa, Beijing, China).

To amplify the sequences of Fiber, Hexon, and Penton, three pairs of oligonucleotide primers were used (Table 1). Amplifications were performed in 50 μL of reaction mixture comprising 2.5 mM dNTPs (4 μL), 10 μM of each primer (2 μL), 500 ng DNA template, and PrimerSTAR HotStart DNA polymerase (TaKaRa Biotechnology Co., Ltd., Dalian, China). Sequence amplification was performed under the following conditions: initial denaturation at 94°C for 3 min, followed by 30 cycles of 94°C for 30 s, 56°C for 1 min, 72°C for 1–3 min for different length of gene targets, and a final extension at 72°C for 10 min in an automated thermal cycler. All the purified PCR products were cloned into a pMD18-T easy vector (TaKaRa Biotechnology Co., Ltd.) for Sanger sequencing (Syn-Biotechnology, Suzhou, China).

**Table 1:** Primers and expected amplicons sizes for detection and amplification.

**Primer**	**Sequence**	**Expected size**
CAdV12-HAF^a^	CGCGCTGAACATTACTACCTTGTC	508 or 1,030 bp
CAdV12-HAR^a^	CCTAGAGCACTTCGTGTCCGCTT	
CAdV2-penFF	AATGGAGTTTTCGTCGTCTCCT	1,500 bp
CAdV2-penRR	TTAGTCGGTGATATTAAGATGGC	
CAdV2-fibFF	GGAATTCATGAAGCGGACACGAAGAGC	1,650 bp
CAdV2-fibRR	CTCGAGTTATTGATTTTCGCCTACATAGGTAAAGG	
CAdV2-hexFF	GGAATTCATGGCAACCCCGTCGATG	2,800 bp
CAdV2-hexRR	CCTCGAGTTAGGTGGTGGCGTTGCC	

a *CAdV12-HAF and CAdV12-HAR primers were used for differentiating CAdV-1 from CAdV-2 (24)*.

### Sequence Alignment and Phylogenetic Analysis

The contig assemblies of Fiber, Hexon, and Penton of CAdV-2 were arranged using the DNASTAR 7.0 software (DNASTAR, Inc., USA) and were compared with those of reference CAdV strains obtained from GenBank. Information of the reference strains is presented in Table 2. The amino acid mutation points of three gene segments were displayed using the MegAlign software. A divergence analysis of the Fiber protein of CAdV-2 was performed with WebLogo (http://weblogo.threeplusone.com/) for sequence logo generator.

**Table 2:** Reference strains used in the study.

**Strain**	**Place of isolation**	**Genotype**	**Submission date**	**Accession no.**
A26/61	Toronto	CAdV-2	Nov-2-96	U77082
India2006	India	CAdV-2	Jul-7-06	DQ839392
CC0710	Jilin, China	CAdV-2	Jun-3-09	GQ241864
CC0710QZ	Jilin, China	CAdV-2	Jun-3-08	EU794687
CC0710QB	Jilin, China	CAdV-2	May-13-08	EU717145
YCA-18	Jilin, China	CAdV-2	Mar-20-07	EF508034
SH01	Shanghai, China	CAdV-2	Oct-10-13	KF727562
CCC-V6	Jilin, China	CAdV-1	Apr-16-07	EF559262
CLL	Toronto	CAdV-1	Apr-14-96	U55001
GLAXO	Toronto	CAdV-1	Jul-26-16	M60937
RI261	UK	CAdV-1	Sep-3-96	Y07760
India2007	India	CAdV-1	Jan-5-07	EF206692
Fox-466-2017-ITA	Italy	CAdV-1	May-29-18	MH399790
574-2013-RS	Italy	CAdV-1	Feb-19-15	KP840549
113-5L	Italy	CAdV-1	Feb-19-15	KP840545
417-2013-L	Italy	CAdV-1	Feb-19-15	KP840547
wolf-835-2015-FRA	France	CAdV-1	Mar-11-18	MH048659
ILT2015	Italy	CAdV-1	Jul-12-16	KX545420
PPV1	Germany	Bat adenovirus	Jul-13-11	JN252129
TJM	Wuhan, China	Bat adenovirus	Nov-23-09	NC016895
Mm32	Japan	Bat adenovirus	May-23-18	LC385828
Skunk adenovirus	USA	Skunk adenovirus	Aug-17-15	NC027708

A phylogenetic tree of the CAdVs identified in this study and other reference canine adenovirus strains was constructed based on nucleotide alignments using the maximum likelihood (ML) method and the Molecular Evolutionary Genetics Analysis software (MEGA X). A bootstrap analysis was performed using 1,000 replications, and the phylogenetic tree was visualized using Tree-view (http://www.evolgenius.info/evolview/).

### Prediction and Analysis of a Hyper-Mutated Fiber Protein Model

A divergence analysis of the Fiber protein of CAdV-2 was performed with WebLogo (http://weblogo.threeplusone.com/). For the protein encoded by *Fiber* that is partly associated with the receptor binding of virus-infected cells, it is necessary to analyze the mutation sites in the model. The Fiber protein sequence of the CAdV-2 reference strain CC0710 in China was downloaded from GenBank, and the model was predicted using the *Fiber* amino acid sequence as a template. Amino acid site mutation was identified in 19 isolates, and the tertiary structure model of Fiber was established using the online software SWISS-MODEL (https://swissmodel.expasy.org/interactive). After prediction, the protein analysis software PyMOL was used for image processing and data analysis of the prediction model.

### Statistical Analysis

The association among clinical signs and presence of the virus in the samples of healthy dogs and dogs with evidence of diarrhea were evaluated using the chi-squared test. Logistic regression was used to identify possible bivariate associations between the presence of CAdV-2 DNA and the presence of other pathogens in the samples. Data were processed using SPSS 19.0 software for Windows (SPSS Inc.; Chicago, IL, USA). A *p* < 0.05 was considered statistically significant.

## Results

### Detection and Analysis of CAdV Co-infection

**Figure 1:**
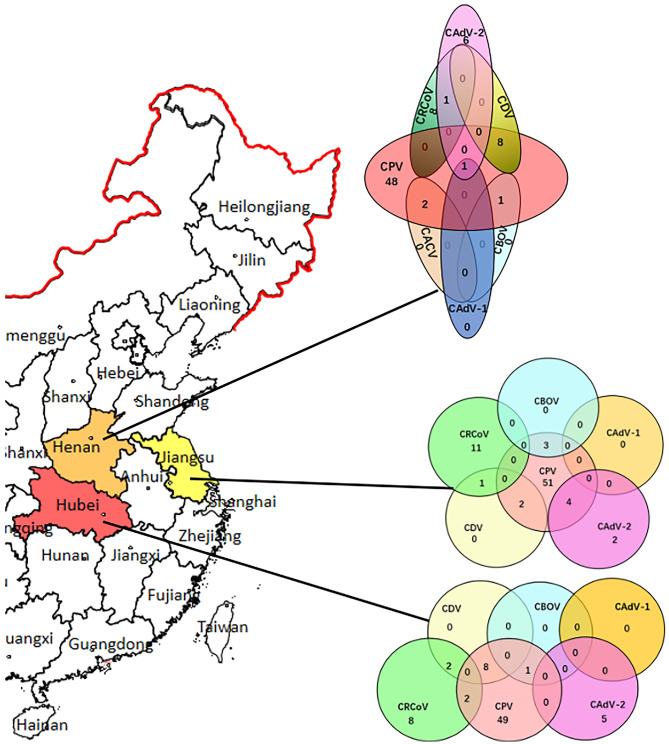
CAdV distribution in China and the infection rates and co-infection status of CPV-2, CDV, CBOV, CRCoV, and CAdV in the Henan, Hubei, and Jiangsu provinces.

CAdV-2 was detected in the samples of 19 dogs with evidence of diarrhea (8.5%, 19/224). The screening data for pathogens present in the 224 rectal swabs were also analyzed to determine the complex co-infection status. The results demonstrated that there were 38 samples with co-infection (16.9%, 38/224), indicating that co-infection was universal, whereas 13 CAdV-2 positive samples has single infection. Among these 38 co-infection samples, co-infection of CAdV-2 and other pathogens accounted for 15.8% (6/38). Of the co-infections of CAdV and other pathogens, co-infections with two pathogens (CPV-2 + CAdV-2, *n* = 4; CRCoV+ CAdV-2, *n* = 1) and three pathogens (CPV-2 + CAdV-1 + CAdV-2, *n* = 1) were identified. Of the 224 diarrhea samples, CPV-2 was found in 80.35% (180/224), CACV in 0.89% (2/224), CBOV in 2.23% (5/224), CDV in 9.82% (22/224), CRCoV in 14.73% (33/224). The results of the pathogen screening are shown in Figure 1. Furthermore, none of the pathogen was detected in the healthy dogs.

A statistically significant difference (*p* < 0.05) could be detected compared the dogs with evidence of diarrhea with health dogs. Logistic regression analysis was performed to evaluate possible bivariate association between the presence of co-infection of CadV-2 and other pathogens with the diarrhea signs, and showed no association between the presence of the CadV-2 and clinical signs (*p* > 0.05).

### Sequence Analysis and Mutation Sites

The Penton, Hexon, and Fiber sequences of the CAdV-2 strains identified in this study are deposited in GenBank, and the accession numbers of each gene fragments are shown in Table 3. Sequence analysis based on the CAdV-2 strains and other reference CAdV strains from GenBank demonstrated that the Penton of the 19 CAdV-2 strains identified herein was 1,434 nucleotides (bp), in length, whereas Fiber was 1,629 bp and Hexon is 2,718 bp in length.

**Table 3:** Relative information of the CAdV-2 strains identified in China in this study.

**Strain**	**Site**	**Date of sampling**	**Age of dog (month)**	**Penton**	**Hexon**	**Fiber**
CH-HN-1701	Nanyang	Jul-3-17	1	MN334535	MN402893	MN599069
CH-HN-1801	Nanyang	Sep-4-18	4	MN334536	MN402894	MN599070
CH-HN-1802	Anyang	Jul-8-18	6	MN334537	MN402895	MN599071
CH-HN-1803	Anyang	Aug-2-18	3	MN334538	MN402896	MN599072
CH-HN-1901	Zhengzhou	Jun-3-19	3	MN334539	MN402897	MN599073
CH-HN-1902	Zhengzhou	Jul-4-19	5	MN334540	MN402898	MN599074
CH-HN-1903	Xinyang	Jul-9-19	4	MN334541	MN402899	MN599075
CH-HN-1904	Xinyang	Aug-3-19	2	MN334542	MN402900	MN599076
CH-HB-1701	Xiangyang	Aug-11-17	5	MN334543	MN402901	MN599077
CH-HB-1801	Xiangyang	Jun-12-18	2	MN334544	MN402902	MN599078
CH-HB-1802	Yichang	Jul-11-18	2	MN334545	MN402903	MN599079
CH-HB-1803	Wuhan	Aug-12-18	1.5	MN334546	MN402904	MN599080
CH-HB-1901	Wuhan	May-3-19	1	MN334547	MN402905	MN599081
CH-JS-1701	Nanjing	Jun-2-17	3	MN334548	MN402906	MN599082
CH-JS-1801	Nantong	Jan-3-18	2	MN334549	MN402907	MN599083
CH-JS-1802	Suzhou	Aug-5-18	3	MN334550	MN402908	MN599084
CH-JS-1803	Changzhou	Aug-01-18	2	MN334551	MN402909	MN599085
CH-JS-1901	Suqian	Jun-11-19	1.5	MN334552	MN402910	MN599086
CH-JS-1902	Huaian	Jul-12-19	1	MN334553	MN402911	MN599087

Sequence comparisons revealed that the Penton of the 19 CAdV-2 strains identified in this study showed 99.5–99.9% nucleotide identity and 99.5–99.9% amino acid identity. Amino acid mutations were the same as those of amino acid sites such as Val18Ala, Ser20Pro, Leu22Pro, Asn38His, and Ile57Thr. Mutations at Gly32Glu were found in two CAdV-2 strains identified here (CH-HB-1801 and CH-JS-1701). At the 471 locus, the reference strain A26/61 was found to be different from all the isolated strains, which had Val at the 471 locus; however, the A26/61 strain had Ala.

Hexon showed 98.1–99.8% nucleotide identity and 98.1–99.9% amino acid identity. Compared with the reference strain A26/61, most of the strains identified here had mutations at a single amino acid site. For example, Asn300Thr and Ais492Arg were harbored in CH-HN-1701. However, specific mutation regions were also identified. Discontinuous mutations occurred between sites 580 and 660 in the CH-HN-1901 strain, whereas other strains did not show mutations at amino acid sites within this range.

**Figure 2:**
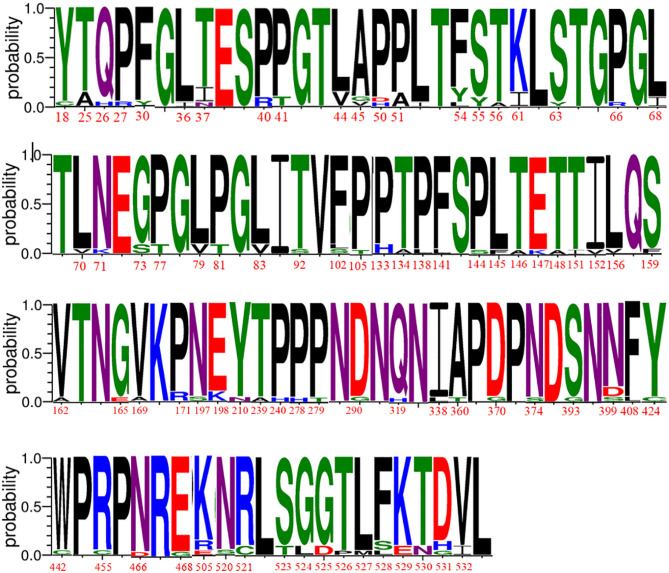
Divergence analysis of fiber proteins of CAdV-2 strains identified in our study.

Fiber showed 97.4–99.8% nucleotide identity and 94.1–99.3% amino acid identity. Compared with that observed for the vaccine strain CLL, the nucleotide and amino acid identity of 19 isolated strains with Fiber was only 79.0–80.5% and 77.3–80.5%, respectively. Among them, the CH-JS-1801 strain shared a high degree of amino acid identity with the Chinese reference strain CAdV-2 CC0710QB. Conversely, alignment of the amino acid sequence of Fiber with the sequences of the reference strains indicated that the variation of the 19 isolated strains is complex. A total of 20 mutations occurred at the CH-HN-1901 amino acid sites, among which 6 were the same as those at the CH-HN-1701 amino acid sites; however, only 12 amino acid sites showed mutations in the CH-HN-1701 strain. The mutation sites of only five tested strains were the same as those of the reference strain CC0710 isolated in Shanghai, China, indicating a large difference in mutation sites. Except for the fact that the amino acids of CH-HN-1903 and CH-HN-1904 are basically the same, all the additional strains have 1- to 20-point mutations. Furthermore, both CH-HN-1701 and CH-HN-1801 had a mutation that occurred at site 25, where the mutation rate for Thr-25 mutation to Ala-25 was 10.5% (two strains in the Henan Province), and it was the same as that observed for CC0710. The main amino acid mutations of fiber proteins were displayed in Figure 2 (Amino acid mutation sites of each strain were displayed detailedly in Table S1).

### Phylogenetic Analysis of Penton, Hexon, and Fiber

**Figure 3:**
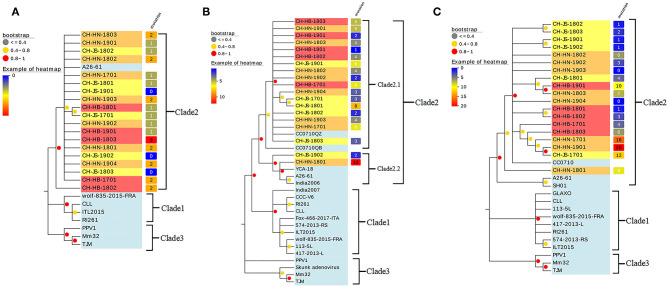
Phylogenetic tree constructed using the complete sequences of **(A)** penton, **(B)** hexon, and **(C)** fiber of canine and bat adenoviruses. The phylogenetic tree was constructed using nucleotide sequences generated in this study and the sequences of CAdV-1, CAdV-2, and bat adenovirus reference strains obtained from GenBank. Bar, numbers of mutation site. Bootstrap values of >80.0% calculated from 1,000 replicates are indicated on the respective branches. Orange background: strains isolated from Henan Province; yellow background: strains isolated from Jiangsu Province; red background: strains isolated from Hubei Province; light blue background: reference strain. The number of mutations is counted in comparison with the A26/61 strain.

Phylogenetic tree and cluster information for the Penton, Hexon, and Fiber nucleotide sequences are shown in Figure 3.

According to the phylogenetic analysis of Penton, 19 CAdV-2 mainly belonged to the type 2 clade and were further divided into three subsets. Among them, the strains aligned with the Canadian A26/61 reference strain belonged to a small branch mainly from the Henan and Jiangsu provinces. However, the CH-JS-1701 and CH-HB-1801 strains were located on a small branch alone, suggesting that these two strains have a similar origin. There was no significant regional difference among the strains.

As shown in Figure 3B, the phylogenetic tree constructed according to the Hexon sequence showed that the strain was divided into three branches, that 19 isolated strains belonged to CAdV-2, and that the other reference CAdV-1 strains were located on an independent branch. Among them, 18 strains were closely related to the reference strains isolated from Jilin Province, whereas the CH-HN-1801 strain was similar to the strains isolated from Canada and India. Hexon Following this definition, the CAdV-2 strains were subdivided into CAdV-2.1 and CAdV-2.2 genotypes. Overall, the two strains CH-JS-1901, and CH-HN-1801 were distant from the other strains in the phylogenetic tree. Considering the geographical distribution, the strains isolated from the Henan and Hebei provinces were clustered on the same branch, whereas those isolated from Jiangsu Province were scattered among the branches.

The phylogenetic tree constructed according to Fiber sequences showed that the CH-HN-1801 strain was clustered with the SH01 and A26-61 strains on a small branch, and the remaining strains were located in another small branch. Isolated strains of Henan Province CH-HN-1802, CH-HN-1902, and Jiangsu Province isolate strains in the evolutionary tree distribution distance is relatively close, CH-JS-1701, and Henan Province isolate strains clustered on another small branch of the evolutionary tree. However, most of the strains were close to the reference strain CC0710 and far from the SH01 and A26-61 strains. The specific distributions are shown in Figure 3C.

### Protein Structure Prediction

**Figure 4:**
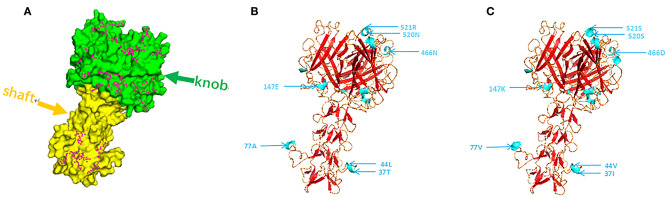
**(A)** Using the fiber protein sequence of the CAdV-2 reference strain, the purple dot was identified as the mutation site of the fiber protein in the 19 strains tested in this study. **(B,C)** Tertiary structure of the Fiber protein of CC0710 strain and synthesis CC0710 strain harbored the major mutations. Altered antigenic epitopes are indicated in blue.

CAdV-2 binds to cell surface receptors through the Fiber protein. As shown in Figure 4, most of the amino acid mutation sites of the 19 isolated strains are concentrated on the knob; the shaft, which comprises proline and hydrophobic amino acid residues, was not conservative, which is associated with the antigen specificity of distinct adenoviruses serotypes. Based on the composition of the fibrillar structural functional regions of human adenoviruses, we found that the structural functional regions of CAdV-2 are composed of amino acids 1–42, 43–365, and 366–542, which are located in the tail, shaft, and head regions, respectively (28–30). The mutant amino acids in the 19 CAdV-2 strains were located at sites 18, 25, 26, 30, 36, 37, 40, and 41, which were located in the tail of the fibrillar process; sites 44, 45, 50, etc., which were located on the basal axis of the fibrillar process, also known as the shaft; and sites 370, 374, 393, etc., which were located in the ganglion region of Fiber, also known as the knob (Figure 4A). There are four differences between Figures 4B,C at sites 37, 77, 408, 520, 521, 523-532 of Fiber.

## Discussion

The present study reports the findings of investigations on the presence of CAdV-2 in 224 dogs from the Henan, Hubei, and Jiangsu provinces, China, from 2017 to 2019. All the dogs presented with diarrheal symptoms, with no obvious respiratory problems, and the prevalence of CAdV-2 infection (8.5%, 19/224 dogs) was verified; CAdV-2 infection was often concomitant with CPV-2 and other pathogenic infections. Even though the CAdV-2 was only detected in dogs with diarrheal symptoms, logistic regression analysis showed no association between the presence of the CAdV-2 and clinical signs. Previous studies have reported the infection of CAdV-2 in dogs with diarrhea, and the number of cases of CAdV-2 infection has gradually increase (17, 19). We speculated the poor association between the presence of the CAdV-2 and diarrhea may derive from small quantity of cases singly positive for CAdV-2. Therefore, whether variant CAdV-2 caused canine enteritis required larger investigation studies and animal regression with CAdV-2 challenge experiments.

Penton, Hexon, and Fiber are the three main structural proteins of CAdV-2. Penton serves as a pedestal, which helps the virus adhere to the cell surface (31, 32), and its sequence is relatively conservative compared with the sequences of reference strains. In the present study, we found that only few amino acid sites on Penton differed from each other, suggesting an important role of Penton in the stability of virus survival function.

Previous study have suggested that the specific epitopes present on the Hexon protein are the target of neutralizing antibodies and the most sensitive part of adenoviruses to immune selection pressure and that its specificity is likely determined by the amino acid sequence of the primary structure of the Hexon protein (33). A comparison between the amino acid sequences of Hexon showed that the continuous site mutations of CH-HB-1701 and the discontinuous site mutations of CH-HN-1901 were first identified in the CAdV-2 isolates, which were different compared with the CAdV-1 reference strains isolated in Europe (8, 9, 11). Whether these continuous site mutations cause substantial changes in antigenic epitopes needs to be further analyzed. The phylogenetic tree based on *Hexon* gene sequences indicated new genotypes for CAdV-2.1 and CAdV-2.2. Even though the 19 CAdV-2 strains could also been sub-classed as new genotypes based on phylogenetic trees about *Penton* and *Fiber* genes, phylogenetic analysis of *Hexon* gene was choosed to be the genotyping criteria for Hexon was the main protein targeted by the host neutralizing antibody. The *Penton* and *Fiber* genes for the proposed Clade 2.2 strains do not also separate into a distinct sub-clade might cause by the independent evolution of each gene. The relationship between CAdV-2 genotyping and evolution of *Penton* and *Fiber* genes needs deeply discussion in future studies.

Analysis of the Fiber gene sequences revealed the same pattern of amino acid substitutions observed in three strains isolated in this study (CH-HN-1701, CH-HN-1801, and CH-HN-1802) and two previously reported strains isolated from bats (Mm32 and TJM) (33, 34). In particular, these amino acid substitutions occurred at positions 51 and 210, which are different from those observed in other proteins. The properties of 50 amino acids sites in the fiber section of CAdV-2 were altered. Seven mutant amino acids (Pro27Arg, Pro40Arg, Pro50His, Pro66Arg, Pro113His, Pro171Arg, Pro278His) changed their properties from uncharged to acidic; two mutant amino acids (Arg155Cys and Asp290Gly) changed from acidic to polar; one mutant amino acid (Asp519Ala) changed from acidic to medium; and three mutant amino acids (Glu147Lys, Glu198Lys, and Asp531His) changed from basic to acidic, and two mutant amino acids (Lys505Glu and Lys529Glu) changed from acidic to basic. The length of the basal axis of adenoviruses has been shown to play a very important role in viral infection and affinity (35). The conserved amino acid region of the fibrillar process is the tail, which is the subgroup specific antigen of adenoviruses and is connected to the five adjacent Penton base proteins (28). In the present study, a total of 49 sporadic mutant amino acids were found to be located on the basal axis. The amino acid properties of 22 sites did not change and only two amino acid sites showed acid to alkaline changes, which had a limited effect on the variation of the properties of the isolated strains.

The RDP 4.36 software was used to predict the possibility of recombination events in three major capsid proteins of the CAdV-2 but no recombination event was predicted (data was not shown). Analyzing the mutations in the CAdV-2 strain is helpful for screening the vaccine strain used in the present study. At present, most vaccines used for preventing CAdV infection in China are replicas of foreign vaccines (36–38). However, over time, some mutations occur in CAdV-2 and the amino acid sites of these mutations are the same as those of the mutations in the CAdV-1 strain. For instance, a mutation from Asn-71 to Arg-71 occurred in the CH-HN-1901 strain and a mutation from Pro-279 to Thr-279 occurred in the CH-HN-1902 strain. The effect of these site mutations on the genetic evolution of the strain needs to be verified in further experiments.

In conclusion, this study describes the prevalence and co-infection of CAdV-2 in some regions of China, revealed that CAdV-2 may be one of the main pathogens causing canine diarrhea. Novel genotypes of CAdV-2 were analyzed. Among 19 CAdV-2 strains, there were much more mutation sites in Fiber than in Penton and Hexon. Thus, new vaccines should be screened to effectively control the dominant strains and expand the scope of immunization.

## Data Availability Statement

The datasets presented in this study can be found in online repositories. The names of the repository/repositories and accession number(s) can be found in the article/Supplementary Material.

## Ethics Statement

Sample collection protocols were approved by the dog's owner and the South China Agricultural University Committee for Animal Experiments (Approved ID: SYXK-2014-0136, March 25, 2014), and samples were collected in accordance with recommendations of the Guide for the Care and Use of Laboratory Animals of the National Institutes of Health.

## Author Contributions

JJ and XX designed the study and wrote the manuscript. WL and WH performed the sampling and data collection. WL performed the clinical investigations, necropsies, and molecular tests. YK, YB, QX, and LY performed the molecular genetic studies and helped to draft the manuscript. All authors have read and approved the final manuscript.

## Conflict of Interest

The authors declare that the research was conducted in the absence of any commercial or financial relationships that could be construed as a potential conflict of interest.
