# Supplementary material for: Novel Genotype Definition and the First Epidemiological Investigation of Canine Adenovirus Type 2 in Dogs in Central China
Source: Front Vet Sci. 2020 Aug 19;7:534. doi: 10.3389/fvets.2020.00534 (PMC7466760; doi:10.3389/fvets.2020.00534)
Supplement: Supplementary file 1 [file Table_1.docx]

Table S1. Main amino acid mutation sites in the Fiber protein of CAdV-2 in the strains identified in China (“CH-”; this study) and in reference strains

| **Strains** | **Mutation sites: amino acid residue** | | | | | | | | | | | | | |
| --- | --- | --- | --- | --- | --- | --- | --- | --- | --- | --- | --- | --- | --- | --- |
|  | 25 | 37 | 44 | 52 | 54 | 71 | 77 | 147 | 198 | 210 | 399 | 466 | 520 | 521 |
| A26/61 | T | T | L | L | F | N | A | K | E | Y | N | N | N | R |
| SH01 | T | T | L | L | F | N | A | K | E | Y | N | N | N | R |
| CC0710 | A | T | L | L | F | N | A | E | E | Y | N | N | N | R |
| wolf-835-2015-FRA | T | T | L | L | F | K | A | E | G | Y | V | P | F | N |
| RI261 | T | T | L | L | F | K | A | E | G | Y | V | P | F | N |
| ILT2015 | T | T | L | L | F | K | A | E | G | Y | V | P | F | N |
| GLAXO | T | T | L | L | F | K | A | E | G | Y | V | P | F | N |
| CLL | T | T | L | L | F | K | A | E | G | Y | V | P | F | N |
| 574-2013-RS | T | T | L | L | F | K | A | E | G | Y | V | P | F | N |
| 417-2013-L | T | T | L | L | F | K | A | E | G | Y | V | P | F | N |
| 113-5L | T | T | L | L | F | K | A | E | G | Y | V | P | F | N |
| TJM | T | T | L | I | F | A | A | A | L | P | A | S | S | A |
| PPV1 | T | T | L | L | F | Q | V | D | G | P | I | P | T | T |
| Mm32 | T | T | L | I | F | A | A | A | L | P | A | S | S | R |
| CH-HN-1701 | A | I | V | I | Y | N | V | E | E | Y | N | N | S | R |
| CH-HN-1801 | A | T | L | L | F | N | A | K | E | Y | N | N | N | R |
| CH-HN-1802 | T | T | L | L | F | N | A | E | E | Y | N | N | N | R |
| CH-HN-1803 | T | T | L | L | F | N | A | E | E | Y | N | N | N | C |
| CH-HN-1901 | T | I | V | L | Y | K | V | E | E | Y | N | N | N | R |
| CH-HN-1902 | T | T | L | L | F | N | A | E | E | Y | N | N | N | R |
| CH-HN-1903 | T | T | L | L | F | N | A | E | E | Y | N | N | N | R |
| CH-HN-1904 | T | T | L | L | F | N | A | E | E | Y | N | N | N | R |
| CH-HB-1701 | T | N | L | L | F | N | A | E | E | Y | N | N | N | R |
| CH-HB-1801 | T | T | L | L | F | N | A | E | E | Y | N | N | N | R |
| CH-HB-1802 | T | T | L | L | F | N | A | E | E | Y | N | N | N | R |
| CH-HB-1803 | T | I | V | L | L | N | A | E | E | Y | N | N | N | R |
| CH-HB-1901 | T | T | L | L | F | N | A | E | E | Y | N | D | N | C |
| CH-JS-1701 | T | T | L | L | Y | N | V | E | E | N | N | N | N | R |
| CH-JS-1801 | T | T | L | L | F | N | A | E | E | Y | N | N | N | R |
| CH-JS-1802 | T | T | L | L | F | N | A | E | K | Y | N | N | N | R |
| CH-JS-1803 | T | T | L | L | F | N | A | E | K | Y | N | N | N | R |
| CH-JS-1901 | T | T | L | L | F | N | A | E | E | Y | D | N | N | R |
| CH-JS-1902 | T | T | L | L | F | N | A | E | E | Y | D | N | N | R |
